# Aortic Syndrome in an Elderly Female: A Case of Type A Intramural Hematoma

**DOI:** 10.1155/crcc/5588181

**Published:** 2025-10-22

**Authors:** Nismat Javed, Shoaib Ashraf, Ankita Gore, Venkata SriRamani Peesapati, Sanjana Narasimhadevara, Karthickrajkumar Kariamanickam, Marin Nicu, Preeti Jadhav, Nassim Krim

**Affiliations:** ^1^ Department of Internal Medicine, BronxCare Health System, Bronx, New York, USA, bronxcare.org; ^2^ Department of Cardiology, Mount Sinai Morningside–BronxCare Health System, Bronx, New York, USA

**Keywords:** aortic syndrome, elderly female, intramural hematoma, prognosis, treatment

## Abstract

Aortic intramural hematoma is a significant presentation of aortic syndromes. It is characterized by bleeding within the aortic media without an intimal defect and can lead to acute aortic dissection if the intimal layer ruptures. Diagnosis requires a high level of suspicion, with CT angiography being essential for confirmation, especially to differentiate it from other conditions. In this case report, we discuss the presentation of an 84‐year‐old African American female with hypothyroidism and hypertension who presented with chest pain, nausea, and hypertension. Lab tests showed mild lactic acidosis and elevated troponins, with a positive drug screen for amphetamines, and she was diagnosed with acute intramural hematoma as well as pericardial effusion on imaging. Aortic intramural hematoma affects elderly individuals with risk factors for severe atherosclerotic disease. Diagnosis involves imaging studies, particularly noncontrast CT scans, followed by contrast CT scans. The prognosis varies based on the type of intramural hematoma. Further research is needed to guide conservative treatment strategies.

## 1. Introduction

Aortic intramural hematoma (IMH) is the second most common presentation of aortic syndromes, with an estimated incidence of 1.98/100,000 person years [1]. Additional estimates state an incidence of 3.5%–28.3% within Type A aortic syndromes [2]. IMH is defined as a distinct lesion characterized by crescentic or circumferential thickening of the aortic wall in the absence of an intimal defect [2]. Chest pain is one of the symptoms of aortic IMH [3]. The pathology of both diseases is slightly different; the initial event in IMH is the rupture of the vasa vasorum, leading to bleeding within the aortic media. If the intimal layer ruptures, known as the entry tear, IMH can progress to acute aortic dissection, potentially marking the beginning of an aortic dissection [4]. The presence of an entry tear is a key diagnostic feature of acute aortic dissection, which usually develops spontaneously rather than from an IMH [4]. Diagnosis requires a high level of suspicion, as clinical presentation can vary, and imaging techniques are essential for confirmation, specifically CT angiography to differentiate IMH from other entities [3, 4]. In this case report, we discuss the clinical presentation of an 84‐year‐old female with radiating chest pain who was diagnosed with aortic IMH on imaging.

## 2. Case Presentation

An 84‐year‐old African American female presented to the emergency department with a 10‐h history of chest pain that was midsternal, radiating to the upper back, and worsened upon exertion, associated with nausea. Vitals on presentation were significant for hypertension (144/101 mm Hg). Physical examination was unremarkable. The patient had a prior history of hypothyroidism and hypertension. She was also a former smoker. The differentials included acute coronary syndrome, aortic dissection, and pancreatitis.

**Figure 1:**
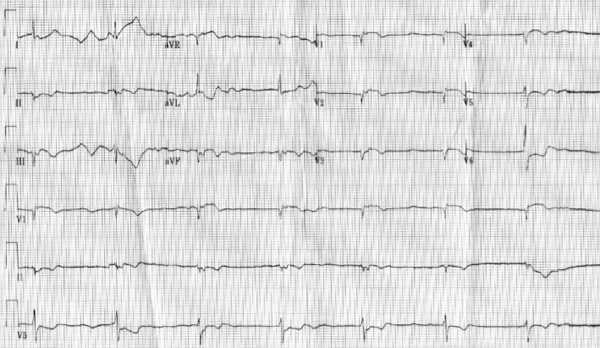
Electrocardiogram on presentation revealed junctional rhythm, T‐wave inversions in V2–V6, and ST‐depressions in V4–V6.

Initial lab investigations were significant for mild lactic acidosis (2.5 mmol/L; reference range: 0.5–1.6 mmol/L) and elevated troponins (44–213 ng/L; reference range: < 12 ng/L). The drug screen returned positive for amphetamines. Imaging on presentation, including chest X‐ray, was normal. Electrocardiogram on presentation revealed junctional rhythm, T‐wave inversions in V2–V6, and ST‐depressions in V4–V6 (Figure 1).

Cardiac critical care unit (CCU) was consulted for concerns about acute non–ST‐elevation myocardial infarction (NSTEMI) and admitted. The thrombolysis in myocardial infarction (TIMI) score on the presentation was 4.

**Figure 2:**
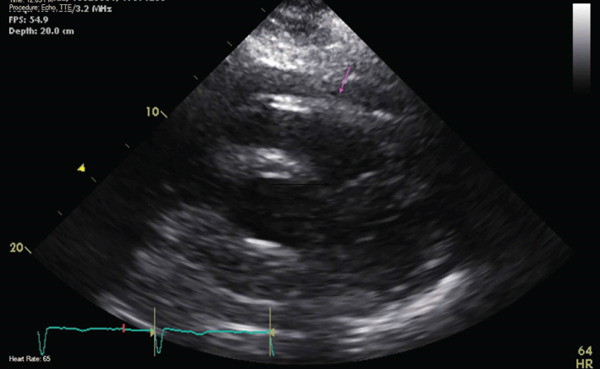
Echocardiogram showing moderate pericardial effusion and hyperdynamic left ventricle as pointed out by red arrows.

The patient underwent an urgent coronary angiogram, which showed patent coronaries with luminal variations, dilated aorta, and suggestive of hyperdynamic left ventricular function. Subsequently, echocardiogram revealed concentric left ventricular hypertrophy, Grade 2 diastolic dysfunction, mildly dilated left atrium, dilatation of aortic root, and ascending aorta. Remarkable abnormalities included a thickening in the proximal ascending aortic wall suggestive of IMH and moderate fluid pericardial effusion (Figure 2).

**Figure 3:**
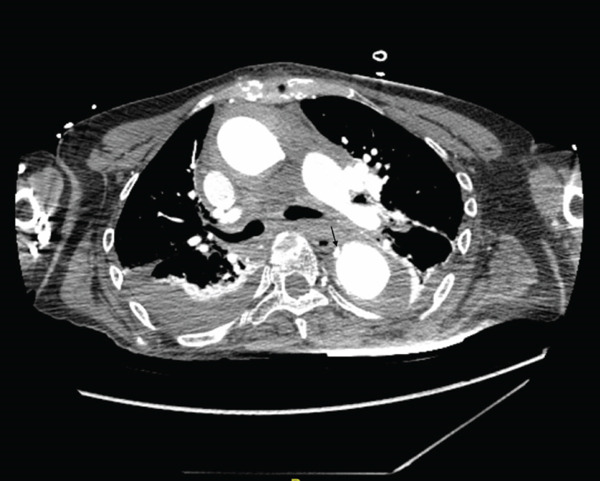
CT findings showing focal small outpouching at the left lateral wall of the ascending thoracic aorta, aortic intramural hematoma at the diaphragmatic hiatus and infrarenal abdominal aorta, hemopericardium measuring up to 15 mm in thickness and small layering bilateral pleural effusions.

Further imaging was also pursued, given IMH. CT chest, abdomen, and pelvis with contrast (Figure 3) confirmed IMH that extended throughout her ascending aorta, arch, and descending aorta.

There was also a moderate pericardial effusion. Her hospital course was complicated by altered mental status and lethargy on presentation.

Given these findings, she was urgently transferred to a tertiary center for emergent cardiothoracic surgical intervention. She underwent a median sternotomy for the replacement of the ascending aorta and hemiarch under circulatory arrest and moderate hypothermia with antegrade cerebral perfusion via the left carotid. Intraoperative findings included a massive IMH starting from a penetrating ulcer on the posterior aspect of the ascending aorta and sinotubular junction, extending proximally to the innominate artery. Her postoperative course was uncomplicated. A repeat echocardiogram after 4 days of the procedure showed a left ventricular ejection fraction of 55% with a normally functioning aortic valve. She was discharged with physical therapy and outpatient follow‐up in the clinic after 3 months.

## 3. Discussion

Aortic IMH is mostly prevalent in elderly individuals with a median age varying from 58 to 71 years (median: 68 years) [4]. Our patient, although slightly older, represented a similar cohort. Additionally, the disease is more common in males [5].

Based on the International Registry of Aortic Dissection, acute IMH accounts for 5.7% of acute aortic syndrome cases [6]. It may possess comparable rates of illness and death to those associated with aortic dissection [6]. The rupture of vasa vasorum in the absence of an intimal tear leads to predisposition of thrombus development that presents as IMH [6]. The development of the disease is considerably higher in the case of risk factors predisposing to severe atherosclerotic disease, specifically poorly controlled hypertension [7]. However, the role of connective tissue disease as a risk factor is still under investigation [7]. In our case, amphetamine use could have been a possible factor suggested by hypertension on presentation [8]. Previous case reports have reported amphetamine use as a possible cause for aortic dissection, but the development of IMH is rare [8]. The postulated pathogenesis includes either the development of autoimmune mechanisms or sympathomimetic impact [8]. An additional entity that can mimic acute coronary syndrome is painless aortic dissection, predominantly in elderly males. In one case, routine evaluation revealed a large ascending aortic aneurysm (75 mm) with an intimal flap, indicating an aortic dissection, and newly developed severe aortic regurgitation. Urgent transesophageal echocardiography confirmed a dissection originating near the left coronary sinus [9]. Similar findings were noted in the case of an African American male [10].

Clinical features causing suspicion for IMH include chest pain and back pain radiating to the back; in a few studies, it has been noted to be significantly associated with Type B IMH [3]. The other clinical features also depend upon the territories involved in the extension of the hematoma [3].

Diagnosis primarily involves imaging studies. On a noncontrast CT scan, acute IMH typically appears as a constant high attenuation (differentiate from dissection) (60–70 HU) thickening of the aortic wall due to an intramural thrombus [6]. This thickening, traditionally crescent‐shaped, can affect a short segment of the aorta or extend along most of its length [6]. After intravenous contrast, the hematoma becomes less noticeable against the contrast‐enhanced aortic lumen [6]. Therefore, patients suspected of acute aortic syndrome should initially undergo nonenhanced multidetector CT imaging to detect minor areas of intramural hyperdensity, which may be hidden after intravenous contrast enhancement [6]. In some cases of IMH, focal contrast enhancement within the thickened aortic wall may be present, possibly due to ulcer‐like projections, penetrating atherosclerotic ulcers from the aortic lumen, or intramural blood pools linked with aortic branch arteries [6]. High‐risk features for complications include ulcer projections, aneurysmal dilatations, hematomal thickness > 16 mm, and intramural blood pools [6]. As observed in our patient, pericardial effusion was also a high‐risk feature on imaging; however, hemopericardium associated with tamponade led to potentially worsened outcomes [11]. While transthoracic echocardiography is extremely useful at detecting pericardial effusion, it can be limited by many factors [3]. While MRI is also another important clinical study, it is limited to resource‐extensive settings and can be time consuming [3].

Hemodynamically unstable patients typically require urgent surgery [11]. Also, complications like cardiac tamponade, pericardial effusion, aortic insufficiency, contained rupture, periaortic hematoma, and malperfusion from branch vessel occlusion necessitate surgical intervention [2]. Acute IMH patients can be periodically imaged to monitor these complications [2]. However, for hemodynamically stable and uncomplicated IMH, certain indicators suggesting a need for surgery have been identified, including wall thickness more than 11 mm, maximal ascending aortic diameter over 48–55 mm, and ulcer‐like projections, all associated with poor outcomes [2]. These high‐risk factors may indicate the need for planned, nonemergency surgery [2]. The debate over conservative medical management for stable, uncomplicated IMH without high‐risk features will likely need more studies examining the natural progression of the disease [2]. In cases of painless aortic dissections, surgery remains the mainstay of treatment [9, 10]. While some data suggest the risk of rupture, aneurysm formation, and sudden death are too high to adopt a conservative strategy, other data suggest a benign disease course that regresses over time [2].

Prognosis depends on the type of aortic IMH [12]. While imaging studies showing the absence of a direct‐flow communication through an intimal tear or disappearance of the hematoma altogether are good prognostic signs for Type B IMH, the prognosis for Type A IMH is controversial, and literature regarding the correlation of imaging features with prognosis is limited [12].

## 4. Conclusions

Aortic IMH predominately affects elderly individuals, especially males, and in individuals with risk factors for severe atherosclerotic disease. Diagnosis involves imaging studies, particularly noncontrast CT scans, followed by contrast CT scans. However, a high degree of suspicion is needed to understand these cases and intervene timely as to prevent poor outcomes. Further studies need to be performed to determine parameters for the conservative mode of treatment.

**Nomenclature**
IMH: intramural hematoma
CCU: cardiac critical care unit
NSTEMI: non–ST‐elevation myocardial infarction
TIMI: thrombolysis in myocardial infarction

## Consent

The patient’s consent was obtained prior to the write‐up of the manuscript and submission.

## Disclosure

All authors agreed to be responsible for the manuscript in its entirety.

## Conflicts of Interest

The authors declare no conflicts of interest.

## Author Contributions

N.J., S.A., A.G., and P.J. conceptualized the manuscript. N.J. performed data curation. M.N., P.J., and N.K. used resources. M.N., P.J., and N.K. supervised the project as well as validated the findings. N.J., S.A., A.G., V.S.P., S.N., and K.K. all contributed to writing the initial draft. N.J., S.A., A.G., V.S.P., S.N., K.K., M.N., P.J., and N.K. reviewed the draft for submission.

## Funding

No funding was received for this manuscript.

## Data Availability

The data that support the findings of this study are available from the corresponding author upon reasonable request.
